# An observational study of emergency department utilization among enrollees of Minnesota Health Care Programs: financial and non-financial barriers have different associations

**DOI:** 10.1186/1472-6963-14-62

**Published:** 2014-02-08

**Authors:** Nathan D Shippee, Tetyana P Shippee, Erik P Hess, Timothy J Beebe

**Affiliations:** 1 Division of Health Policy and Management, University of Minnesota, MMC 729, 420 Delaware Street SE, Minneapolis, MN 55455, USA; 2 Department of Emergency Medicine, Mayo Clinic, 200 First Street SW, Rochester, MN 55905, USA; 3 Division of Health Care Policy and Research, Mayo Clinic, 200 First Street SW, Rochester, MN 55905, USA

## Abstract

**Background:**

Emergency department (ED) use is costly, and especially frequent among publicly insured populations in the US, who also disproportionately encounter financial (cost/coverage-related) and non-financial/practical barriers to care. The present study examines the distinct associations financial and non-financial barriers to care have with patterns of ED use among a publicly insured population.

**Methods:**

This observational study uses linked administrative-survey data for enrollees of Minnesota Health Care Programs to examine patterns in ED use—specifically, enrollee self-report of the ED as usual source of care, and past-year count of 0, 1, or 2+ ED visits from administrative data. Main independent variables included a count of seven enrollee-reported financial concerns about healthcare costs and coverage, and a count of seven enrollee-reported non-financial, practical barriers to access (e.g., limited office hours, problems with childcare). Covariates included health, health care, and demographic measures.

**Results:**

In multivariate regression models, only financial concerns were positively associated with reporting ED as usual source of care, but only non-financial barriers were significantly associated with greater ED visits. Regression-adjusted values indicated notable differences in ED visits by number of non-financial barriers: zero non-financial barriers meant an adjusted 78% chance of having zero ED visits (95% C.I.: 70.5%-85.5%), 15.9% chance of 1(95% C.I.: 10.4%-21.3%), and 6.2% chance (95% C.I.: 3.5%-8.8%) of 2+ visits, whereas having all seven non-financial barriers meant a 48.2% adjusted chance of zero visits (95% C.I.: 30.9%-65.6%), 31.8% chance of 1 visit (95% C.I.: 24.2%-39.5%), and 20% chance (95% C.I.: 8.4%-31.6%) of 2+ visits.

**Conclusions:**

Financial barriers were associated with identifying the ED as one’s usual source of care but non-financial barriers were associated with actual ED visits. Outreach/literacy efforts may help reduce reliance on/perception of ED as usual source of care, whereas improved targeting/availability of covered services may help curb frequent actual visits, among publicly insured individuals.

## Background

In their role within the health care safety net, emergency departments (EDs) provide an important service to the public through 24-hour per day operations and identification and stabilization of patients with acute, life threatening conditions [1]. ED use is especially frequent among publicly insured and low-income populations [2]–[4], who disproportionately face worries about costs and coverage limitations and non-financial barriers such as lack of personal transportation and child care [4,5]. Problems with access to primary care and concerns about costs, even for those who have health insurance, may be associated with more ED visits and poorer health for patients who visit the ED [6]. For public insurance programs, barriers that persist even in the face of health coverage represent a key target for improved services and outreach. This is especially important considering that a decreasing number of EDs are experiencing an increasing number of visits, and that ED visit rates for those covered by Medicaid account for much of the overall increase [7,8].

Expansion of health coverage (e.g., under the Affordable Care Act) might help limit high ED utilization by improving insured access to primary care and other providers [9]. However, coverage is not equivalent to access [10]. Rather, an entire array of non-coverage related barriers to access—including office hours/availability, appointment problems, wait times, transportation, and lack of a usual primary care provider—likely drive ED visits and reliance on ED as a proxy source of primary care [11]–[15]. Moreover, given common perceptions of the ED as free or less expensive than regular care [2,13] patients’ concerns about costs and coverage—which do not necessarily align with actual coverage status [16]—may also contribute to ED utilization patterns [17].

To better understand how financial and non-financial factors may be associated with frequent ED utilization among publicly insured patients, the present study assesses the associations that financial concerns about healthcare costs and coverage and practical/non-financial access barriers to care have with publicly insured individuals’ identification of the ED as usual source of care, and the actual number of ED visits. Measuring these associations increases our understanding of what may and may not work in seeking to address shortcomings in outreach, availability, and other persistent challenges that may be associated with reliance on, and frequent use of, ED services among publicly insured individuals.

## Methods

### Data

This cross-sectional study is a secondary analysis of administrative and survey data from adult participants in the 2008 study, “Disparities and Barriers to Utilization among Minnesota Health Care Program Enrollees”; overall survey results were reported previously [18]. The original 2008 study, an update and partial replication of a similar 2003 effort [19], was funded by the Minnesota Department of Human Services and sought to identify problems with access, barriers, and self-reported utilization among publicly insured individuals in Minnesota. Administratively derived measures, for July 1, 2007 - June 30, 2008, pertained to selected health conditions and utilization, whereas survey data, collected July-December 2008, covered self-reported attitudes, utilization, access, and barriers to care.

### Study sample

The study sample included non-institutionalized enrollees of Minnesota Health Care Programs (Minnesota’s Medicaid/CHIP and low-income public health insurance programs). MHCP includes three main programs: Medical Assistance (MA, Minnesota’s Medicaid program), General Assistance Medical Care (GAMC), a state-funded program covering low-income individuals (mainly adult men) not eligible for MA, and MinnesotaCare, a state and federally subsidized program for children and adults without insurance who are not eligible for MA or GAMC. The original study used a stratified design to oversample minorities and obtain comparable proportions of racial/ethnic groups. The survey was mailed, with telephone follow-ups for initial non-response offered in various languages as needed. Survey response rate was 44% in the study overall. The original study received approval from the Institutional Review Boards (IRBs) of Minnesota Department of Human Services and the University of Minnesota; the present project also has review clearance (defined “not human subjects research,” as a secondary analysis of de-identified data) from the Mayo Clinic and University of Minnesota IRBs.

### Dependent variables

Our two dependent variables were “ED as usual provider” and number of ED visits. *ED as usual provider* was a dichotomous measure; the survey item asked where the individual “Which of the following places best describes where you usually go for your health care?,” with emergency department listed alongside seven other choices including primary care providers’ offices, urgent care, and an open-ended option for respondents to identify anything else. For this study, we coded the variable as 1 = ED as usual source of care and 0 = not. The second variable, ED visits, came from an administrative data count of ED visits for each enrollee over the past year. We coded ED visits as the following: 0 = no visits; 1 = 1 visit; 2 = 2+ visits. In sensitivity analyses, we modeled ED visits in various ways: as a binary (none versus any visits), a differently aggregated ordinal (0, 1–2, and 3+), or an uncategorized count. Most of these analyses produced substantively similar results, and so we used the 0-1-2+ coding based on a review of the variable’s natural distribution among this sample, the need to distinguish between single-visit users and other users, and high zero inflation which complicated count models.

### Independent variables

Our key independent variables concerned financial and non-financial barriers to accessing health care from survey data (the question and items are shown, along with the rest of the survey, as an appendix to the original report [18]). Barrier-related items were developed primarily based on focus group data and involvement of community members in the original studies [18,19] and a local survey as part of another project [20]. Respondents were asked whether each in a series of items was a problem in getting the health care they needed. *Financial barriers* represents a set of seven self-reported items pertaining to financial cost and coverage concerns, most closely representing Penchansky and Thomas’ “affordability” aspects of access [21]. These financial barriers included worries that: insurance won’t cover care, that the respondent will have to pay more than expected, that he/she will have to pay more than he/she can afford, that medications will cost too much, not being sure about being dropped from the public health care program, not knowing what the health plan covers, and not knowing where to go with questions about coverage. The survey asked whether each barrier was ‘a big problem,’ ‘a small problem,’ or ‘not a problem.’ Big and small problem were combined to indicate each financial concern. We used these measures to construct a dummy variable indicating *any* financial concern (1 = yes; 0 = no) and as a summed count of financial concerns (range: 0–7).

*Non-financial barriers* included seven practical, non-financial barriers to access (“non-financial barriers”), best understood as representative of accessibility and accommodation under the Penchansky and Thomas model [21]. Rather than coverage or payment, these self-reported barriers concerned practical hardships which complicated access to care, including: transportation difficulties, problems making appointments, not knowing where to go for care, work/family responsibilities, offices/clinics not being open at suitable times, obtaining childcare, and not being able to utilize one’s preferred provider. Items were coded similarly to financial concerns; again, we constructed a dummy variable for *any* non-financial barrier (1 = yes; 0 = no) and a summed count of barriers (range: 0–7).

#### Covariates

Because differences in health status may explain some variations in ED use among vulnerable populations [22,23] we tested available binary indicators for International Classification of Diseases-9th revision (ICD-9-CM) diagnostic categories derived from administrative data. We included two indicators with significant effects: one for the presence of a mental health disorder (ICD-9-CM codes 290–329), and another for an injury or poisoning (codes 800–999), as a first-listed diagnosis. We did not include a third significant category (codes 780–799) because it pertained to general or ill-defined symptoms and conditions or abnormal test results, and had no clear interpretation, whereas the association of mental health problems and with more frequent ED use is supported by research literature [24]–[27] and injury/poisoning (e.g., broken bones, trauma, accidental poisonings) is an expected class of reasons for ED use. Also, because ED use could simply be an expression of a tendency for higher health care utilization overall [27], we included count of primary care office visits (capped at 25 to limit outlier effects) and an indicator for whether the enrollee had an inpatient stay from administrative data. Other covariates included age; education (1–8, with 4 indicating a high school degree and 7 representing a four-year college degree); being married or “living with a partner in a marriage-like relationship” (versus single/divorced/widowed); being employed (versus not); birth in the United States; and indicators for race/ethnicity (Black, Hispanic/Latino, Native American/Alaska Native, and Asian; “White, non-Hispanic” was the reference category). Race/ethnicity was based on administrative data initially; we used self-reported race from surveys to correct or fill in these values. Previous work has shown strong concordance between self-reported and administrative race/ethnicity in this population [28].

### Analyses

Analyses were completed using StataSE 12 [29]. For all results shown, we used weights to correct for unequal selection probabilities and to ensure that the sample characteristics matched those of underlying population (i.e., non-institutionalized MHCP enrollees as of March 31, 2008).

Following descriptive statistics, we employed multivariate logistic regression to assess the associations of key independent variables with enrollee-reported ED as usual source of care. Next, we employed ordinal logistic regression to assess the associations of the same independent variables with 0, 1, or 2+ ED visits; we also added ED as usual source of care as a covariate to examine and control for people’s self-reported care tendencies (significance of other covariates did not change based on the inclusion or exclusion of this variable, however). Following both models, to improve interpretability for combinations of predictors, we used post-estimation predicted probabilities [30], based on regression-adjusted estimates.

We performed supplemental sensitivity analyses to assess the robustness of findings under alternative modeling strategies. First, regarding missing data, most variables had no or less than 2% missing; the only ones with higher missing were ED as usual provider (5.86%), and counts of financial (6%) and non-financial (5.95%) barriers. However, to address the effects of any larger amounts of missing and test robustness of findings, we first completed analyses using simple list-wise deletion, then completed sensitivity analyses employing multiple imputation using chained equations (MICE) for all missing data. Substantive results differed very little; in particular, findings for key independent variables (financial and non-financial barriers) remained the same. Therefore, we present the unimputed (yet still weighted) results here for the effective/non-missing sample of 1,737 individuals, but said results are robust to multiple imputation. Second, because ED as usual source of care was a relatively rare outcome and sample size was moderately small, we completed sensitivity analyses to adjust for possible bias. Specifically, this entailed the Firth method [31] to penalized maximum likelihood (“firthlogit” command in Stata, similar to King and Zeng’s proposed approach [32]); significance levels did not change, and odds ratios were within .01 of uncorrected estimates, indicating relatively limited bias in our estimates. Finally, due to sample size concerns and limited information on enrollment, we did not limit or exclude participants based on months of MHCP enrollment; in other words, administratively derived measures may or may not be misrepresentative due to lapses in coverage. As such, we performed sensitivity analyses with the basic indicators of enrollment by month that we did have. Most enrollees had 11 or 12 months of enrollment (73.68% of the effective sample of 1,737); this increased to 79.3% with 10 or more months (or 77.6% if limited to at least 10 *consecutive* months), and 93.2% with at least six months of enrollment (90.9% if limited to at least six *consecutive* months). When we limited analysis of ED visits to those with six or more (n = 1,579) and 10 or more consecutive months (n = 1,347) of enrollment, non-financial barriers remained significant in ordered logit models, as did all administratively derived diagnosis and utilization measures.

## Results

Table 1 displays weighted descriptive statistics. Among adult enrollees, 1.5% reported ED as usual source of care (95% CI: 8.7%-22.2%); 70.6% had no ED visits in the past year (95% C.I.: 66.99%-74.3%), 17.1% had 1 (95% C.I.: 14.1%-20.2%), and 12.3% had 2+ visits (95% C.I.: 9.7%-14.8%). Nearly three quarters (72.8%, 95% C.I.: 69.1%-76.5%) reported at least one financial concern (with an average of 2.9 concerns reported, 95% C.I.: 2.7-3.1); 61.7% (95% C.I.: 57.6%-65.7%) reported at least one non-financial barrier, with an average of 1.6 barriers (95% C.I.: 1.5-1.7). For diagnoses, 29% (95% C.I.: 25.4%-32.7%) and 28.3% (95% C.I.: 24.5%-32.1%) had a mental health or injury/poisoning diagnosis, respectively, for the year. Enrollees had an average of 4.98 (95% C.I.: 4.6-5.4) primary care office visits; 17.8% (95% C.I.: 14.7%-12.8%) had an inpatient stay for the year. Enrollees were, on average, 41.7 years old (95% C.I.: 40.3-43.0), 66.2% female (95% C.I.: 62.2%-70.2%), with a median education of high school diploma, 84.3% U.S.-born (95% C.I.: 81.9%-86.8%), 40.3% married (95% C.I.: 36.2%-44.5%), 39.9% employed (95% C.I.: 35.7%-43.99%), and 66.1% White (95% C.I.: 63.3%-68.95%).

**Table 1:** Weighted descriptive statistics for adult enrollees of Health Care Programs

	**Mean**	**Percent**	**[95% C.I.]**	
ED as usual source of care		1.54%	0.87%	2.22%
ED visits in past year				
No ED visits		70.64%	66.99%	74.31%
1 ED visit		17.11%	14.05%	20.16%
2+ ED visits		12.25%	9.70%	14.79%
Any financial concern		72.84%	69.14%	76.54%
Count of financial concerns	2.92		2.71	3.12
Any non-financial barrier		61.67%	57.62%	65.73%
Count of practical barriers	1.58		1.45	1.72
Mental health diagnosis		29.02%	25.36%	32.68%
Injury or poisoning diagnosis		28.31%	24.50%	32.13%
Office visits in past year	4.98		4.57	5.38
Inpatient stay in past year (yes/no)		17.74%	14.66%	20.81%
Age	41.66		40.29	43.03
Female		66.18%	62.18%	70.19%
Education	4.59		4.47	4.70
Never attended school		2.44%	1.90%	2.98%
8th grade or less		5.10%	3.67%	6.52%
Some high school		15.24%	12.42%	18.06%
High school diploma/GED		31.78%	27.88%	35.68%
Technical/vocational school		11.04%	8.27%	13.81%
Some college/Associate's degree		24.38%	20.77%	28.00%
Four-year college degree		8.61%	6.12%	11.10%
Graduate/professional degree		1.41%	0.43%	2.39%
Born in the U.S.		84.32%	81.86%	86.77%
Married		40.32%	36.19%	44.45%
Employed		39.85%	35.70%	43.99%
White, non-Hispanic		66.13%	63.32%	68.95%
Black		14.27%	12.99%	15.56%
Hispanic/Latino		4.86%	4.00%	5.71%
Asian		7.24%	5.09%	9.39%
American Indian/Native American		6.94%	5.59%	8.29%

N = 1737.

Notes: Analyses are weighted to account for sampling design.

Multivariate analyses (Table 2) revealed that both financial concerns and non-financial barriers significantly and positively predicted reporting ED as usual source of care, but in the full model (Model 3), the association of non-financial barriers with ED as usual source of care was statistically mediated (p = .580; this occurred regardless of strategy for missing data/imputation, suggesting robustness). Financial barriers were still associated with greater likelihood (OR = 1.4, 95% C.I.: 1.03-1.8). An injury or poisoning diagnosis was associated with greater odds (OR = 3.23, 95% C.I. 1.01-10.28), and every office visit was associated with lower likelihood (OR = 0.76, 95% C.I.: 0.64-0.92), of identifying ED as usual provider. Compared to White, non-Hispanic individuals, black individuals had 10.7 times the odds (95% C.I.: 2.7-42.7) and Native American individuals had 6.6 times the odds (95% C.I.: 1.7-24.9) of reporting ED as usual source of care. Other variables showed no significant relationships.

**Table 2:** Logistic regression of reporting ED as usual source of care on independent variables among adult Minnesota Health Care Programs enrollees

	**Model 1**			**Model 2**			**Model 3**		
	**OR**	**p**	**95% C.I.**	**OR**	**p**	**95% C.I.**	**OR**	**p**	**95% C.I.**
Financial concerns	1.389	0.010	1.080-1.785				1.358	0.032	1.026-1.798
Non-financial barriers				1.306	0.007	1.075-1.586	1.063	0.580	0.857-1.318
Mental health diagnosis	1.019	0.966	0.424-2.451	0.920	0.862	0.360-2.349	0.973	0.953	0.395-2.398
Injury or poisoning	3.194	0.049	1.007-10.130	3.037	0.064	0.937-9.843	3.229	0.047	1.014-10.280
# Office visits	0.763	0.004	0.635-0.916	0.773	0.006	0.642-0.929	0.765	0.005	0.635-0.921
Inpatient stay	1.574	0.352	0.605-4.095	1.446	0.461	0.543-3.850	1.550	0.365	0.601-3.999
Age	1.011	0.359	0.988-1.035	1.011	0.299	0.990-1.033	1.012	0.336	0.988-1.036
Female	1.522	0.389	0.585-3.959	1.287	0.597	0.505-3.280	1.485	0.421	0.566-3.897
Education	0.859	0.498	0.554-1.334	0.849	0.506	0.523-1.376	0.852	0.462	0.555-1.307
Married	2.050	0.212	0.663-6.338	1.932	0.241	0.642-5.813	2.096	0.182	0.707-6.212
Employed	0.438	0.082	0.173-1.109	0.459	0.084	0.190-1.110	0.432	0.078	0.170-1.097
Born in U.S.	0.617	0.350	0.225-1.697	0.652	0.411	0.235-1.810	0.622	0.359	0.226-1.715
White, non-Hispanic									
Black	10.989	0.001	2.739-44.092	10.211	0.003	2.224-46.891	10.726	0.001	2.692-42.740
Hispanic/Latino	1.696	0.620	0.210-13.713	1.752	0.632	0.176-17.422	1.676	0.629	0.206-13.596
Asian	0.681	0.712	0.089-5.234	0.689	0.720	0.090-5.293	0.645	0.672	0.085-4.924
American Indian/Alaska Native	6.697	0.006	1.731-25.915	6.704	0.008	1.645-27.320	6.578	0.006	1.741-24.855
Constant	0.002	0.000	0.000-0.022	0.005	0.000	0.000-0.077	0.002	0.000	0.000-0.022

N = 1,737.

Note: Analyses are weighted to account for sampling design.

In ordinal logistic regression models for number of ED visits from administrative data (Table 3), financial concerns were not significant in any model (e.g., p = 0.219 in the full model, Model 3). However, even beyond the significant associations shown for ED as usual source of care and administratively-derived diagnosis and utilization measures, and controlling for demographics, each additional non-financial barrier was associated with 0.21 greater odds of being in a higher category of ED use (95% C.I.: 1.05-1.40).

**Table 3:** Ordinal logistic regression of 0, 1, or 2+ ED visits in past year on independent variables among adult enrollees of Minnesota Health Care Programs

	**Model 1**			**Model 2**			**Model 3**		
	**OR**	**p**	**95% C.I.**	**OR**	**p**	**95% C.I.**	**OR**	**p**	**95% C.I.**
Financial concerns	1.003	0.950	0.926-1.086				0.939	0.219	0.849-1.038
Non-financial barriers				1.155	0.013	1.031-1.294	1.210	0.009	1.048-1.398
ED as usual provider	4.864	0.001	1.924-12.300	4.563	0.001	1.812-11.496	4.921	0.001	1.879-12.888
Mental health diagnosis	2.288	0.000	1.506-3.476	2.142	0.000	1.399-3.280	2.148	0.000	1.402-3.291
Injury or poisoning	4.562	0.000	2.974-6.997	4.843	0.000	3.133-7.487	4.806	0.000	3.106-7.437
# Office visits	1.081	0.000	1.040-1.123	1.081	0.000	1.040-1.123	1.080	0.000	1.040-1.123
# Inpatient stays	3.697	0.000	2.302-5.940	3.926	0.000	2.451-6.287	3.932	0.000	2.467-6.266
Age	0.994	0.337	0.983-1.006	0.996	0.490	0.984-1.008	0.996	0.519	0.984-1.008
Female	1.016	0.942	0.654-1.580	0.984	0.943	0.632-1.531	0.970	0.892	0.624-1.507
Education	0.900	0.091	0.797-1.017	0.892	0.059	0.791-1.004	0.892	0.061	0.792-1.005
Married	1.315	0.234	0.838-2.062	1.263	0.316	0.800-1.996	1.265	0.314	0.800-2.002
Employed	1.058	0.808	0.671-1.667	1.034	0.887	0.655-1.630	1.052	0.827	0.667-1.659
Born in U.S.	1.319	0.311	0.772-2.251	1.355	0.289	0.772-2.378	1.336	0.304	0.769-2.319
White, non-Hispanic									
Black	2.129	0.001	1.357-3.339	2.036	0.002	1.295-3.202	2.078	0.002	1.318-3.275
Hispanic/Latino	2.018	0.053	0.991-4.110	2.018	0.055	0.985-4.137	2.095	0.043	1.025-4.285
Asian	0.678	0.449	0.248-1.854	0.597	0.351	0.202-1.766	0.630	0.403	0.213-1.861
American Indian/Alaska Native	1.872	0.011	1.155-3.033	1.689	0.037	1.032-2.764	1.712	0.030	1.054-2.782
Cut-point 1	9.085	0.000	3.214-25.680	11.275	0.000	3.893-32.658	10.217	0.000	3.518-29.676
Cut-point 2	38.614	0.000	13.436-110.978	48.347	0.000	16.487-141.772	43.989	0.000	14.879-130.051

N = 1737.

Note: Analyses are weighted to account for sampling design.

**Figure 1:**
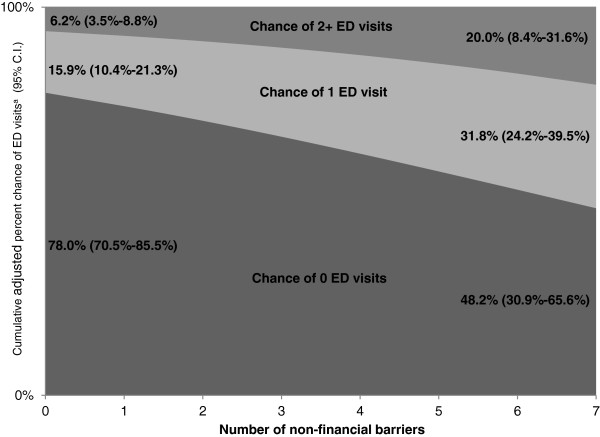
**Adjusted chance of 0, 1, or 2+ ED visits, by count of non-financial barriers.** X axis: Number of non-financial barriers (0–7) Y axis: Cumulative adjusted percent chance of ED visits^a^ (95% C.I.). Figure shows adjusted percent chance of number of ED visits (0, 1, or 2+ visits) by number of non-financial access barriers; chances are cumulative (i.e., totaling to 100%). At zero non-financial barriers, the predicted chances of 0, 1, or 2+ ED visits are 78% (95% C.I.: 70.5%-85.5%), 15.9% (95% C.I.: 10.4%-21.3%), and 6.2% (95% C.I.: 3.5%-8.8%), respectively. The percent chance for 0 ED visits is lower, and those for 1 or 2+ ED visits are higher, across higher counts of non-financial barriers. Under the condition of having all seven non-financial barriers, the chances of 0, 1, or 2+ ED visits are, respectively, 48.2% (95% C.I.: 30.9%-65.6%), 31.8% (95% C.I.: 24.2%-39.5%), and 20% (95% C.I.: 8.4%-31.6%). ^a^Represents predicted probabilities for each outcome, based on estimates from ordinal logistic regression as shown in Table 3.

For better interpretation, we used estimates from the full ordinal logistic regression to calculate predicted probabilities of each level of ED use (Figure 1). Under conditions of no non-financial barriers (and controlling for all other variables), chance of zero ED visits was 78.0% (95% C.I.: 70.5%-85.5%), chance of 1 visit was 15.9% (95% C.I.: 10.4%-21.3%), and chance of 2+ visits was 6.2% (95% C.I.: 3.5%-8.8%). However, for all seven non-financial barriers, the chance of zero ED visits was lower, at 48.2% (95% C.I.: 30.9%-65.6%), whereas the chances of 1 and 2+ visits were 31.8% (95% C.I.: 24.2%-39.5%) and 20.0% (95% C.I.: 8.4%-31.6%), respectively.

## Discussion

Using administrative and survey data from a diverse sample of publicly insured adults in Minnesota, we analyzed the associations that financial concerns and non-financial access barriers had with identification of ED as usual source of care and actual ED visits. Financial concerns and non-financial barriers had different associations: after controlling for each other and covariates, robust associations remained mainly between: 1) financial concerns and identification of ED as usual source of care; and 2) non-financial access barriers and actual ED visits (from administrative data). The implications of this study are that policies which address financial concerns may decrease the already-low percentage of the publicly insured population that reports the ED as their usual source of care, but that limiting frequent actual ED use requires addressing the non-financial barriers that complicate non-emergent outpatient care.

Self-reported concerns and barriers among enrollees of public insurance programs, while previously identified as contributing to patterns of ED use [13,14,17], are actually associated with different challenges to reaching and accessing health care. On the one hand, financial concerns that exist despite Minnesota offering relatively generous coverage (compared to other states) [33] suggest a potential lack of awareness and knowledge of available services and costs. These financial concerns also may represent a lack of faith or trust that regular care is feasible—a belief that, in the current findings, coincides robustly with individuals’ belief that the ED is the go-to source for care. In an alternative formulation, it could be argued that concerns about coverage and identification of ED as usual source of care capture similar latent phenomena among publicly insured individuals’ worldview—an anxiety or distrust regarding regular services and reliance on putatively free ED services. Financial concerns also may reflect an actual awareness of specific financial disincentives for non-emergency use of the ED that currently exist in Minnesota’s public health care programs (copayments currently range from $3.50 upwards, depending on the program in which they are enrolled), although this is speculative and we are not aware of the extent to which the definition of emergency medical conditions is enforced.

In comparison, non-financial access barriers represent individual reports of experienced difficulty, often due to logistical, time- or resource-related constraints. While their relationship with reporting ED as usual source of care was statistically mediated by financial concerns, these non-financial access barriers were strongly associated with actual ED visits—similar to previous literature [13,14], but here even controlling for individuals’ naming of ED as usual source of care, the presence of a mental health and injury or poisoning diagnosis, and office visit and inpatient stay utilization. This suggests that, rather than being associated with ED use simply as a correlate or expression of financial vulnerability, or indirectly as associated with poorer mental health, trauma, or broader utilization patterns, such non-financial barriers indicate practical difficulties that are embedded in the tangible constraints embedded in individuals’ everyday lives (e.g., child care, transportation, office hours, and others). It also suggests that enrollees’ financial concerns do not associate directly with ED use.

Together, findings suggest that coverage expansion (under PPACA or similar programs) will fail to address frequent ED use unless such expansion is paired some key efforts. These include outreach and education in helping enrollees understand what is available to them in non-ED care. Lack of knowledge about available services is a key concern for Minnesota’s publicly insured population, particularly those facing language and cultural barriers [34]. Unfortunately, county governments in Minnesota appear to shoulder much of the burden of outreach, but their financial capacity for outreach is already limited [35,36], and it appears new PCACA provisions dedicated to improving health literacy may be poorly funded [37]. Moreover, as noted above, financial concerns are only associated with reporting the ED as usual source of care, but not actual ED visits. The rate of reporting the ED as usual provider is low (1.5%, which is comparable to previous studies finding between .9% and 4.6%, depending on the population [38,39]). As such, increased awareness of what is covered or not covered may be indirectly related to ED visits (via identification of the ED as the usual provider). More directly, the key issues associated with actual ED visits, namely non-financial barriers, require more pointed efforts, considering that they pertain to accessibility of services (e.g., office hours) and limited everyday resources in one’s personal life (e.g., transportation or child care). As such, helping individuals identify a non-ED usual source of care is likely to be, at best, a half-measure, and at worst, may exacerbate some of the accessibility issues if coverage is expanded under the affordable care act—in other words, without better accessibility of services or consideration of other resources needed in patients’ lives, linking more publicly insured individuals with non-ED providers may be futile or simply stretch *those* providers’ capacity similar to what has happened with EDs. Addressing non-financial barriers requires finding ways to better target services with an eye toward the difficulties people face which are not cost/coverage-related. ED users often rely on hospital outpatient departments, community health centers, and other public health care clinics as a usual source of care [40]. Addressing non-financial barriers to these health care venues—such as expanding after-hours care, improving options for transportation, child care, and family responsibilities, or providing more information about available providers—may represent one strategy for limiting frequent ED use. Without outreach and supportive services, and potentially greater capacity of non-ED providers, coverage may not mean improvements in patterns of ED use.

The findings of this study should be read in light of its limitations. The data are secondary and cross-sectional, and so we are not able to truly test causality. Also, the sample, while diverse, cannot generalize to all publicly insured individuals in the U.S. However, Minnesota provides relatively comprehensive and affordable (e.g., low co-pay) coverage [33], and so findings here may represent a best-case scenario of the challenges that publicly insured individuals encounter. In a separate vein, although administrative data contained demographics and utilization, information on individuals’ incomes, health behaviors, or specific reasons for ED visits were unavailable. In addition, administratively derived variables may be subject to bias due to the fact that, because of sample size concerns, we did not limit analyses based on months of enrollment (which means that estimates may be affected by changes or lapses in enrollment). Sensitivity analyses of ED visit models based on consecutive enrollment showed that non-financial barriers, diagnosis indicators, and utilization measures remained significant, but the potential for bias should not be ignored. Certain survey measures here may be subject to recall bias, or to social desirability bias (e.g., underreporting of the ED as usual provider, or lower reporting of barriers as a problem, if respondents saw these as socially undesirable). Finally, the overall response rate (44%) was moderately low, but not surprising: response rates vary between 12% and 82% [41,42], depending on follow-up efforts, and are often low among Medicaid and low-income populations—e.g., 38% [43] up to, in some cases, 50% [44]. Response rates here varied across adult sampling strata, ranging between 34.7% and 53% [18], and so we controlled for race and other demographics, along with weighting to control for sampling design. However, response rates remain a caveat, as with any study using survey data.

## Conclusions

In sum, financial concerns were solely associated with reporting ED as usual provider, whereas non-financial access barriers were associated with actual ED use. Expanded coverage will not guarantee reductions in frequent ED use. Outreach/literacy efforts may help prevent viewing ED as one’s usual provider; improved availability of non-emergent and publicly covered services, particularly including supportive services such as transportation and child care, may help curb frequent ED visits.

## Abbreviations

ED: Emergency department.

## Competing interests

The authors declare that they have no competing interests.

## Authors’ contributions

NS conceived the study, drafted the manuscript, and performed analyses. TS assisted in revising the manuscript and contributed to analytic design. EH revised the manuscript. TB contributed to study design and assisted in writing and revising the manuscript. All authors read and approved the final manuscript.

## Pre-publication history

The pre-publication history for this paper can be accessed here:

http://www.biomedcentral.com/1472-6963/14/62/prepub
